# Epistatic QTL pairs associated with meat quality and carcass composition traits in a porcine Duroc × Pietrain population

**DOI:** 10.1186/1297-9686-42-39

**Published:** 2010-10-26

**Authors:** Christine Große-Brinkhaus, Elisabeth Jonas, Heiko Buschbell, Chirawath Phatsara, Dawit Tesfaye, Heinz Jüngst, Christian Looft, Karl Schellander, Ernst Tholen

**Affiliations:** 1 Institute of Animal Science, Group of Animal Breeding and Genetics, University of Bonn, Endenicher Allee 15, 53115 Bonn, Germany; 2 ReproGen- Centre for Advanced Technologies in Animal Genetics and Reproduction, Faculty of Veterinary Science, University of Sydney, Australia; 3 Department of Animal and Aquatic Sciences, Faculty of Agriculture, Chiang Mai University, Chiang Mai, Thailand

## Abstract

**Background:**

Quantitative trait loci (QTL) analyses in pig have revealed numerous individual QTL affecting growth, carcass composition, reproduction and meat quality, indicating a complex genetic architecture. In general, statistical QTL models consider only additive and dominance effects and identification of epistatic effects in livestock is not yet widespread. The aim of this study was to identify and characterize epistatic effects between common and novel QTL regions for carcass composition and meat quality traits in pig.

**Methods:**

Five hundred and eighty five F_2 _pigs from a Duroc × Pietrain resource population were genotyped using 131 genetic markers (microsatellites and SNP) spread over the 18 pig autosomes. Phenotypic information for 26 carcass composition and meat quality traits was available for all F_2 _animals. Linkage analysis was performed in a two-step procedure using a maximum likelihood approach implemented in the QxPak program.

**Results:**

A number of interacting QTL was observed for different traits, leading to the identification of a variety of networks among chromosomal regions throughout the porcine genome. We distinguished 17 epistatic QTL pairs for carcass composition and 39 for meat quality traits. These interacting QTL pairs explained up to 8% of the phenotypic variance.

**Conclusions:**

Our findings demonstrate the significance of epistasis in pigs. We have revealed evidence for epistatic relationships between different chromosomal regions, confirmed known QTL loci and connected regions reported in other studies. Considering interactions between loci allowed us to identify several novel QTL and trait-specific relationships of loci within and across chromosomes.

## Background

Until now, most QTL studies have considered additive and dominance effects and sometimes imprinting effects, but epistatic interactions between two or more loci are commonly ignored. The significance of interactions between different loci in explaining the genetic variability of traits has long been controversial.

Epistatic effects can be clearly defined and verified when a combination of two mutations yields an unexpected phenotype that cannot be explained by the independent effect of each mutation [1]. For example, Steiner et al. [2] have demonstrated the effect of gene interactions for a binary expressed trait (coat color), which is influenced by two or three loci. However, the evaluation of epistasis for complex traits is much more demanding because these traits are influenced by environmental effects and large numbers of polymorphic loci [3]. For complex traits, it is useful to analyze the variation in a resource population established for QTL studies, by applying epistatic QTL models.

Most published studies on epistatic effects of interacting QTL have focused on plants and laboratory animals rather than livestock species, which is a paradox since it seems obvious that the variance of a complex trait in livestock animals cannot be explained by additive genetic effects alone [4].

In plants, investigations into epistatic effects concern mainly rice hybrids for traits such as grain yield, plant height and heating date [5,6], but epistatic effects have also been identified in maize, oat and *Arabidopsis *[7].

Most epistatic QTL studies related to mammals analyze data from laboratory animals. Brockmann et al. [8] have shown that in a mouse intercross used to select for body weight and fat accumulation, epistatic effects contributed 33% and 36% of the total phenotypic variation, respectively, whereas epistatic effects contributed only 21% of the variation. Kim et al. [9] have investigated non-insulin-dependent diabetes in two backcross populations of mice i.e. B6 and CAST crosses. They have detected five interacting QTL in the B6 cross but none in the CAST cross. Shimomura et al. [10] have detected ten epistatic QTL connected to circadian behavior in mice. Sugiyama et al. [11] have found six single QTL associated with blood pressure in rats but 36% of this trait's phenotypic variance could be explained by a single two-dimensional epistatic factor. Koller et al. [12] have examined the mineral density of bones in a reciprocal cross in rats and found epistatic effects between known and novel QTL and between pairs of completely unknown QTL.

In livestock species, epistatic effects have been detected in chicken and swine. In chickens, Carlborg et al. [13,14] have identified epistatic effects on growth traits, which accounted for up to 80% of the genetic variation. In swine, ten QTL pairs for eight muscle fiber traits in an intercross between Iberian and Landrace breeds [15] and interacting genomic regions for carcass composition traits and intramuscular fat content in F_2 _crosses between Pietrain and three other commercial lines[16] have been reported. Additional studies have revealed epistatic relationships influencing meat color, fatty acid composition and reproductive traits such as teat number or litter size [17-20].

In this work, we have evaluated the importance of epistatic effects in pig breeding by identifying epistatic QTL effects for carcass composition and meat quality in an F_2 _cross composed of commercial pig lines.

## Methods

### Animals and analyzed traits

In this study, we used 585 F_2 _pigs from 31 full-sib families that were the product of a reciprocal cross of the Duroc and Pietrain (DuPi) breeds. The F_1 _generation was the product of crosses between Duroc boars and Pietrain sows and between Pietrain boars and Duroc sows. All animals were kept at the Frankenforst experimental research farm of the Rheinische Friedrich-Wilhelms-University in Bonn. The phenotypes of all the F_2 _animals were recorded in a commercial abattoir, according to the rules of German performance stations [21]. In total, 13 traits related to carcass composition and 13 traits related to meat quality were analyzed. Table 1 contains an overview and definitions of all the carcass composition and meat quality traits that were analyzed. Intramuscular fat content (IMF) was determined by the Soxhlet extraction method with petroleum ether [22]. More detailed information about the carcass composition and meat quality traits can be found in Liu et al. [23].

**Table 1:** Mean and standard deviation for carcass composition and meat quality

**Traits for carcass composition**^**1**^	Abbreviation	**N**^**2**^	Mean	**SD**^**3**^
Carcass length [cm]	carcass length	585	97.95	2.70
Dressing [%]	dressing	585	76.76	1.93
Backfat shoulder [cm]	BFT-shoulder	585	3.43	0.43
Backfat 13th/14th rib [cm]	BFT-13/14	585	1.64	0.30
Backfat loin [cm]	BFT-loin	585	1.33	0.31
Backfat mean [cm]	BFT-mean	585	2.13	0.31
Backfat thickness above *M. long. dorsi*, 13/14^th ^ribs [cm]	BFT-thickness	585	1.13	0.27
Side fat thickness [cm]	side fat	585	2.72	0.67
Fat area above the *M. long. dorsi *at 13/14^th ^rib [cm^2^]	fat area	585	16.27	2.84
Loin eye area at 13/14^th ^rib, *M. long. dorsi *[cm^2^]	loin eye area	585	51.82	5.37
Ratio of fat to muscle area	Fat muscle ratio	585	0.32	0.06
Estimated carcass lean content, Bonner formula [%]	ECLC	585	58.73	2.42
Estimated belly lean content [%]	EBLC	585	58.16	2.98

**Traits for meat quality 1**				

pH-value *M. long. dorsi *45 min p.m.	pH 1 h loin	585	6.56	0.20
pH-value *M. long. dorsi *24 h p.m.	pH 24 h loin	585	5.51	0.10
pH decline *M. long. dorsi*	pH decline	585	1.05	0.22
pH-value *M. semimembranosus *24 h p.m.	pH 24 h ham	585	5.64	0.13
Conductivity *M. long. dorsi *45 min p.m	cond. 1 h loin	585	4.32	0.62
Conductivity *M. long. dorsi *24 h p.m.	cond. 24 h loin	585	2.79	0.78
Conductivity *M. semimembranosus *24 h p.m.	cond. 24 h ham	585	4.81	2.14
Meat color, opto-value	meat color	585	68.61	5.65

**Traits for meat quality 2**				

Drip loss [g]	drip loss	342	2.12	0.96
Cooking loss [g]	cooking loss	342	24.87	2.22
Thawing loss [g]	thawing loss	342	8.10	1.98
Warner-Bratzler shear force [kg]	shear force	324	35.27	6.62
Intra muscular fat content [%]	IMF	272	6.99	2.37

^1 ^Estimated carcass lean content = 59.704-1.744*(loin eye area)-0.147*(fat area)-1.175*(BFT-sh)-0.378*(side BFT)-1.801*(BFT thickness); estimated belly lean content = 65.942+0.145*(loin eye area)-0.479*(fat area)-1.867*(side BFT)-1.819*(BFT-loin); backfat mean = the average of backfat loin, backfat shoulder and backfat 13^th^/14^th ^rib; dressing: chilled carcass weight relative to live weight at slaughter; fat area [cm^2^] according to Herbst [63]

^2 ^N: number of records

^3 ^SD: standard deviation

### Statistical analyses

One hundred and twenty five microsatellites and six SNP markers were used to genotype animals of the parental (P), F_1 _and F_2 _generations. Genetic markers were equally spaced on the 18 pig autosomes and covered 89% of these. In comparison to Liu et al. [23], who analyzed the data with a single QTL model, 18 genetic markers (microsatellites and SNP) were added to the data set. The CRI-MAP 2.4 software was used with the options "build", "twopoint" and "fixed" to recalculate the sex-average linkage map [24]. Additional information regarding the markers, i.e. genetic position (in Kosambi cM), number of identified alleles and polymorphism information content are given in Additional file 1 (see Additional file 1).

To identify significant environmental effects, the data were analyzed by linear models including a relevant fixed effects model (model 0) as in Liu et al. [23]. All the models contained a polygenic effect (u_k_), which is distributed as N(0, Aσ^2^_u_), where A reflects the numerator relationship matrix and e_ijk _the residual effect:

(0) $${\text{y}}_{\text{ijk}}={\text{ F}}_{\text{i}}+\beta {\text{cov}}_{\text{j}}+{\text{u}}_{\text{k}}{\text{+ e}}_{\text{ijk}}$$

For carcass composition and intramuscular fat content (IMF), the season/year of birth and the sex were included in the model as fixed effects (F) and carcass weight and age at slaughter as covariates (βcov). For traits like pH, conductivity and meat color, factors including sex, slaughter season, carcass weight and age at slaughter were used. Family, sex, carcass weight and age at slaughter were included in the analyses of drip loss, thawing loss, cooking loss and shear force.

Liu et al. [23] had analyzed the data set by the Haley-Knott regression [25], which was extended in this study for the pH decline and IMF traits.

Interactions between two QTL were detected by the series of model comparisons suggested by Estelle et al. [15]. The statistical analysis can be subdivided into the following two steps, which were performed using the statistical package Qxpak 4.0 [26].

#### Step 1: Preselection of epistatic regions

Additive and dominance effects of individual QTL were excluded from the first step of the analysis. To characterize distinguishable genome regions, all chromosomes were separated into 5 cM intervals because of computational limitations.

(1) $$\begin{array}{l} {\text{y}}_{\text{ijk}}={\text{F}}_{\text{i}}+\beta {\text{cov}}_{\text{j}}+\left({\text{c}}_{\text{aa}}{\text{I}}_{\text{aa}}+{\text{c}}_{\text{ad}}{\text{I}}_{\text{ad}}+{\text{c}}_{\text{da}}{\text{I}}_{\text{da}}+{\text{c}}_{\text{dd}}{\text{I}}_{\text{dd}}\right)\;\\ \;\;\;\;\;\;\;\;\;\;+{\text{u}}_{\text{k}}+{\text{e}}_{\text{ijk}} \end{array}$$

Model 1 includes all the possible genetic interactions between pairs of chromosomal segments (I_aa_, I_ad_, I_da _and I_dd_) but does not include the main genetic effects themselves. The regression coefficients c_aa_, c_ad_, c_da _and c_dd _were calculated according to Cockerham's suggestions for epistatic interaction [27]:

$$\begin{array}{l} {\text{c}}_{\text{aa}}={\text{P}}_{\text{1}}\left(\text{QQ}\right){\text{P}}_{\text{2}}\left(\text{QQ}\right)–{\text{P}}_{\text{1}}\left(\text{QQ}\right){\text{P}}_{\text{2}}\left(\text{qq}\right)\\ \;\;\;\;\;\;\;\;–{\text{ P}}_{\text{1}}\left(\text{qq}\right){\text{P}}_{\text{2}}\left(\text{QQ}\right)+{\text{P}}_{\text{1}}\left(\text{qq}\right){\text{P}}_{\text{2}}\left(\text{qq}\right)\\ {\text{c}}_{\text{ad}}={\text{P}}_{\text{1}}\left(\text{QQ}\right){\text{P}}_{\text{2}}\left(\text{Qq}\right)–{\text{P}}_{\text{1}}\left(\text{qq}\right){\text{P}}_{\text{2}}\left(\text{Qq}\right)\\ {\text{c}}_{\text{da}}={\text{P}}_{\text{1}}\left(\text{Qq}\right){\text{P}}_{\text{2}}\left(\text{QQ}\right)–{\text{P}}_{\text{1}}\left(\text{Qq}\right){\text{P}}_{\text{2}}\left(\text{qq}\right)\\ {\text{c}}_{\text{dd}}={\text{P}}_{\text{1}}\left(\text{Qq}\right){\text{P}}_{\text{2}}\left(\text{Qq}\right). \end{array}$$

The definitions of these interaction terms follow the rules of Varona et al. [28]. P_1 _and P_2 _refer to the probability of a QTL at locations 1 and 2, P(QQ) the probability of the grandparental line (Duroc) being homozygous, P(qq) the probability of the other grandparental line (Pietrain) being homozygous and P(Qq) the probability of being heterozygous. These equations imply unlinked interacting loci [29]. The IBD probabilities were computed by a Markov chain Monte Carlo algorithm with 10000 iterations [26]. Model 1 was tested against model 0 with likelihood ratio tests (LRT) to assess the significance of the effects of interacting QTL. Nominal P-values were calculated assuming chi-squared distribution of the LRT with four degrees of freedom. Interacting QTL pairs with a nominal P-value < 0.001 were selected to be further analyzed in step 2.

However, the results of this model comparison cannot be directly used for the detection of epistasis because the two regions might interact solely in an additive way. The exclusion of the main genetic effects and the definition of widely-spaced 5 cM pseudo-loci are justified by the long computing time necessary for this unsaturated genetic model.

In addition to interactions between regions on different chromosomes, intrachromosomal interactions were investigated. To avoid large, overlapping confidence intervals, interacting QTL positions were selected when the genome regions involved were larger than 30 cM. If the two regions are closer than 30 cM, there is a high risk that an interaction might be observed, which can be explained in reality by a single QTL.

#### Step 2: Calculation of epistasis

Purely epistatic effects were quantified by model 2, which covers all possible genetic main effects and interaction effects. A 1-cM scan was performed within 40 intervals of preselected genome regions identified in step 1.

(2) $$\begin{array}{l} {\text{y}}_{\text{ijk}}={\text{F}}_{\text{i}}+\beta {\text{cov}}_{\text{j}}+\left({\text{c}}_{\text{a1}}{\text{a}}_{\text{1}}+{\text{ c}}_{\text{d1}}{\text{d}}_{\text{1}}\right)+\left({\text{c}}_{\text{a2}}{\text{a}}_{\text{2}}+{\text{c}}_{\text{d2}}{\text{d}}_{\text{2}}\right)\\ \;\;+\left({\text{c}}_{\text{aa}}{\text{I}}_{\text{aa}}+{\text{c}}_{\text{ad}}{\text{I}}_{\text{ad}}+{\text{c}}_{\text{da}}{\text{I}}_{\text{da}}+{\text{c}}_{\text{dd}}{\text{I}}_{\text{dd}}\right)+{\text{u}}_{\text{k}}+{\text{e}}_{\text{ijk}} \end{array}$$

The regression coefficients for the main effects of the two individual QTL were defined as:

$$\begin{array}{l} {\text{c}}_{\text{a1}}={\text{P}}_{\text{1}}\left(\text{QQ}\right)–{\text{P}}_{\text{1}}\left(\text{qq}\right)\\ {\text{c}}_{\text{d1}}={\text{P}}_{\text{1}}\left(\text{Qq}\right)\\ {\text{c}}_{\text{a2}}={\text{P}}_{\text{2}}\left(\text{QQ}\right)–{\text{P}}_{\text{2}}\left(\text{qq}\right)\\ {\text{c}}_{\text{d2}}={\text{P}}_{\text{2}}\left(\text{Qq}\right). \end{array}$$

Factor "a" in model 2 is defined as the individual additive effect and "c" is the regression coefficient for the differences in probabilities of being homozygous for alleles of the Duroc grandparental line (QQ) and for alleles of the Pietrain line (qq). A positive additive genetic value would indicate that alleles originating from the Duroc line show a greater effect than alleles from the other parental line and vice versa. The dominance effect "d" is described as a deviation of heterozygous animals from the mean of both types of homozygous individuals. In the case of a positive dominance value, an increase in the trait of interest is the result of a heterozygous genotype.

(3) $$\begin{array}{l} {\text{y}}_{\text{ijk}}={\text{F}}_{\text{i}}+\beta {\text{cov}}_{\text{j}}+\left({\text{c}}_{\text{a1}}{\text{a}}_{\text{1}}+{\text{c}}_{\text{d1}}{\text{d}}_{\text{1}}\right)+\left({\text{c}}_{\text{a2}}{\text{a}}_{\text{2}}+{\text{c}}_{\text{d2}}{\text{d}}_{\text{2}}\right)\\ \;\;+{\text{u}}_{\text{k}}+{\text{e}}_{\text{ijk}} \end{array}$$

Finally, the statistical contrast between models 2 and 3 for evidence of epistasis was carried out using an LRT with four degrees of freedom in the numerator.

As discussed in Mercade et al. [30], permutation techniques cannot be applied here because an infinitesimal genetic value is included. A randomization of the data would destroy the family structure. Nevertheless, it is necessary to prove the reliability of epistatic QTL pairs. For this purpose, a Bonferroni correction assuming statistical independence every 40 cM was used as in Noguera et al. [17]. The genome-wide critical values of LRT for the significance levels associated with type I errors where α = 0.05, 0.01 or 0.001 were 18.00, 20.45 and 26.21, respectively.

To verify the importance of each epistatic interaction effect involved (a × a, a × d, d × a and d × d; a for additive and d for dominance), the simple heuristic method of Estelle et al. [15] was used. This method judges an epistatic effect as relevant (significant) if the effect size exceeds two residual SD of model 0.

The proportion of the phenotypic variance explained by the genetic components was calculated by the differences between the residual variances of the compared models.

## Results

### Step 1: Preselection of QTL pairs

The number of significant QTL pairs identified in step 1 varied from three to 34 for different traits. In general, low numbers were detected for traits that are known to have high measurement errors due to environmental effects (drip loss, cooking loss and thawing loss) or to the error-prone measurement technique (side fat). In this step, all QTL identified as significant in the single-QTL analysis [23] were also found to be significant in combination with other QTL in the bi-dimensional analysis of step 1.

The significant QTL regions identified in step 1 are interesting candidates for epistasis, but the results of this scan cannot be used as final proof for such effects because the main and interactive genetic effects are not separated. For a final validation of epistatic effects, a fully saturated model including genetic main effects and interaction effects is needed, which leads directly to step 2.

### Step 2: Calculation of epistatic effects

In the final step, the epistatic relationship between two QTL was estimated using model 2. Table 2 gives detailed information on all the significant epistatic QTL pairs according to position, the LR-statistics and the proportion of the phenotypic variance explained by the particular pairs of loci. In general, the number of true epistatic QTL pairs was less than the number of preselected pairs of QTL regions. Fifty-six epistatic QTL pairs were identified across the 18 autosomes for 19 different traits. Intrachromosomal epistatic QTL were located on porcine chromosomes SSC5 (*Sus scrofa *chromosome 5), 8 and 17 for IMF, fat area and loin eye area, respectively.

**Table 2:** Evidence of epistatic QTL loci for carcass composition and meat quality traits

Carcass composition	**SSC pos.1 (cM)**^**1**^	**SSC pos. 2 (cM)**^**1**^	**LR**^**2**^	**Epist. Var**^**4**^	**QTL Var**^**5**^
BFT 13/14 rib	16 (80)	18 (21)	22.9**	3.45	4.59

BFT shoulder	2 (207)	15 (84)	20.8**	3.15	4.56
	9 (57)	10 (151)	19.8**	2.99	3.37

BFT thickness	7 (138)	13 (61)	20.9**	3.27	5.16

Dressing	5 (1)	9 (15)	18.5*	2.82	4.17

ECLC	***2 (135)***	4 (98)	19.0*	2.90	4.53
	***2 (125)***	7 (1)	19.4*	2.96	5.04
	***8 (62)***	10 (79)	22.9**	3.49	5.34

Fat area	***6 (112)***	12 (32)	21.0**	3.20	4.13
	6 (73)	13 (11)	19.8*	3.02	5.79
	8 (36)	8 (127)	23.4**	3.55	5.16

Fat muscle ratio	***2 (125)***	7 (1)	30.4***	5.88	5.88
	***8 (62)***	10 (80)	21.6**	2.94	2.94
	8 (80)	17 (45)	19.4*	3.03	5.88

Loin eye area	2 (135)	4 (96)	18.8*	2.87	4.86
	***8 (58)***	10 (70)	24.8**	3.77	6.01
	17 (55)	17 (80)	48.7***	7.26	10.41

**Meat quality 1**	**SSC pos.1 (cM)**^**1**^	**SSC pos.2 (cM)**^**1**^	**LR**^**2**^	**Epist. Var**^**4**^	**QTL Var**^**5**^

pH 1 h loin	2 (156)	18 (9)	18.0*	2.45	3.79
	3 (34)	13 (85)	21.5**	3.14	4.14
	8 (1)	15 (77)	18.1*	2.80	4.14
	12 (45)	16 (1)	26.2***	4.15	4.48

pH 24 h loin	3 (16)	11 (39)	21.0**	4.11	4.11
	4 (14)	11 (16)	39.4***	6.85	6.85
	10 (84)	18 (24)	19.6*	2.78	4.11

pH decline loin	3 (13)	6 (41)	21.5**	3.06	4.64
	3 (52)	18 (22)	20.3**	3.05	4.37
	6 (39)	14 (84)	22.5**	3.31	4.37
	8 (6)	**15 (71)**	18.6*	2.78	4.37
	12 (48)	16 (1)	26.8***	4.11	4.37
	***15 (61)***	17 (29)	19.3*	2.78	4.37

pH 24 h ham	***1 (108)***	5 (126)	26.9***	4.07	12.59
	***2 (179)***	7 (122)	18.1*	2.27	4.44
	7 (88)	12 (1)	24.5**	3.70	3.70
	10 (84)	18 (23)	23.2**	3.76	5.19
	***15 (61)***	18 (92)	27.6***	4.51	5.93

Conductivity 1 h loin	3 (10)	14 (113)	23.8**	3.62	5.16

Conductivity 24 h loin	5 (52)	13 (75)	26.6***	4.04	5.65
	6 (13)	13 (20)	20.5**	3.12	4.77

Conductivity 24 h ham	***10 (99)***	13 (30)	18.4*	2.83	4.05

Meat colour	7 (80)	12 (26)	22.4**	3.41	4.06

**Meat quality 2**	**SSC pos.1 (cM)**^**1**^	**SSC pos.2 (cM)**^**1**^	**LR**^**2**^	**Epist. Var**^**4**^	**QTL Var**^**5**^

Cooking loss	1 (97)	16 (63)	21.3**	5.18	6.41
	2 (186)	15 (16)	21.8**	5.27	6.61
	4 (43)	16 (102)	19.6*	4.77	7.03
	5 (4)	18 (82)	22.2**	5.40	7.89
	7 (50)	13 (13)	18.9*	4.59	7.33
	7 (47)	16 (108)	20.5**	4.96	8.48
	7 (40)	17 (60)	24.2**	5.88	8.69
	8 (85)	18 (8)	31.2***	7.50	10.22

Thawing loss	2 (49)	4 (105)	18.4*	4.48	6.61
	15 (8)	17 (1)	19.1*	4.63	6.52

Shear force	***2 (166)***	7 (87)	19.9*	5.00	9.17
	***2 (150)***	13 (112)	19.2*	4.83	9.00
	***2 (145)***	16 (102)	21.8**	5.47	9.74
	8 (84)	8 (111)	18.7*	4.71	6.54

IMF	1 (263)	6 (101)	23.4**	8.23	10.85
	5 (57)	5 (87)	24.2**	8.52	13.34

SSC *Sus scrofa *chromosome

^1 ^position in Kosambi cM; in bold presented QTL loci have been detected as single QTL by Liu et al. 2007 [23]

^2 ^LR: 2-log likelihood ratio

^3 ^three genome-wide significance levels were used: 0.1% significant value (LR = 26.21, nominal p < 0.0001,***), 1% significant value (LR = 20.45, nominal p < 0.0005,**), 5% suggestive value (LR = 18.00, nominal p < 0.001,*)

^4 ^proportion (%) of phenotypic variance explained by epistasis calculated as the proportion of the residual variances due the epistatic QTL effects on the residual variances excluding the epistatic QTL effects

^5 ^proportion (%) of phenotypic variance explained by both QTL and their interaction term calculated as the proportion of the residual variances due the QTL effects on the residual variances excluding the QTL effects

Overall, 19 a × a, 11 a × d, 13 d × a and 29 d × d significant interactions were observed. For 16 epistatic QTL pairs, it was not possible to detect any more relevant effects (see additional file 2). Although the general epistatic interaction term was significant for 16 QTL pairs, the effect size of the involved single epistatic effects did not exceed two residual SD (model 2).

The proportion of the phenotypic variance explained by the particular interaction term ranged from 2.5% to 8.5%. The proportion of epistatic variance relative to the entire QTL variance exceeded 50% in most cases (Table 2).

### QTL for carcass composition traits

**Figure 1:**
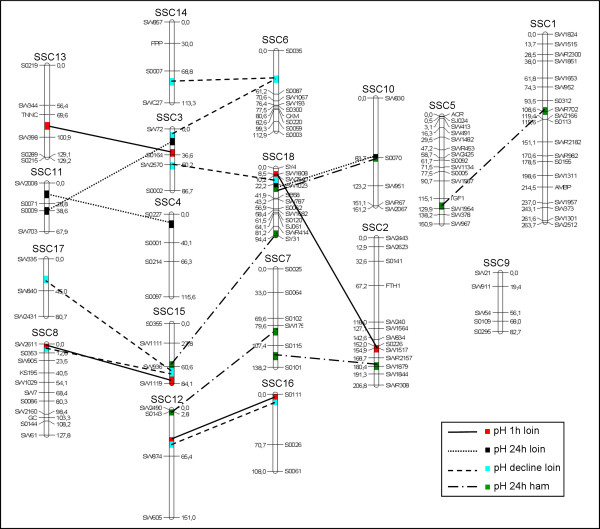
**Epistatic QTL network for pH traits**. Lines represent the epistatic relationship among two loci; different type of lines displays different traits

Seventeen epistatic QTL pairs were detected for seven carcass composition traits. These were located on all autosomes except 1, 4, 11 and 14. The epistatic loci were classified into two highly significant (P < 0.001), nine significant (P < 0.01) and six suggestive (P < 0.05) QTL relationships (Table 2). Chromosomal loci of interest were located on SSC2, SSC4, SSC7, SSC8 and SSC10, where multiple epistatic QTL pairs were detected (Figure 1). Regions located on SSC8 (58 to 62 cM) and SSC10 (70 to 80 cM) showed a significant epistatic interaction for the fat:muscle ratio, the loin eye area and ECLC. The relationship between these two QTL loci explained 3% to 4% of the phenotypic variance of these traits.

Furthermore, high d × d interaction effects were observed for ECLC for one QTL on SSC2 (125 to 135 cM), which interacted with one locus on SSC4 (96 to 98 cM) and another locus on SSC7 (1 cM). Additionally, epistatic QTL pairs were detected for the same loci on SSC2 (135 cM) and SSC4 (96 to 98 cM) related to the loin eye area and also along SSC2 (125 cM) and SSC7 (1 cM) for the fat:muscle ratio. In general, these interacting genomic areas showed the highest d × d interactions in comparison to other single epistatic effects, except the loci on SSC2 and SSC7, where the d × a interaction was the most prevalent. Two to 6% of the phenotypic variance was explained by the relationships between SSC2 and SSC4 and between SSC2 and SSC7 for these carcass composition traits.

No epistatic effects were identified for carcass length, shoulder BFT, mean BFT, side fat and estimated lean belly content.

### QTL for meat quality traits

A total of 14 suggestive (P < 0.05), 18 significant (P < 0.01) and seven highly significant (P < 0.001) QTL were identified for all meat quality traits except drip loss (Table 2). With regard to the number of epistatic QTL pairs, the cooking loss trait involved eight interacting QTL pairs and the pH decline six, which were the highest numbers of epistatic loci for all meat quality traits.

Close relationships were found between SSC8 (1 to 6 cM) and SSC15 (71 to 77 cM) and between SSC12 (45 to 48 cM) and SSC16 (1 cM) for pH 1 h loin and pH decline (Figure 1). For these epistatic effects, a × a and d × d interactions exceeded two SD and were generally more prevalent than a × d or d × a (see Additional file 2). The highest explained proportion of the phenotypic variance was 6.85% for an epistatic QTL pair located on SSC4 (14 cM) and SSC11 (16 cM) related to pH 24 h in loin. The proportion of the phenotypic variance of meat quality traits explained by epistasis ranged from 2.27% to 4.51%. For the measurements of conductivity in loin and ham, four epistatic relationships between seven QTL loci were observed.

Within the group of meat quality traits examined, 16 epistatic relationships among loci were identified (Table 2). For cooking loss, a locus on SSC7 (40 to 50 cM) showed a × d, d × a and d × d interactions with regions on SSC13 (13 cM), SSC16 (108 cM) and SSC17 (60 cM). Additionally, a relationship was identified between the epistatic QTL on SSC16 (102 cM) and one locus on SSC4 (43 cM), but none of the epistatic effects exceeded two SD. The identified loci on SSC4 and SSC7 in combination had no significant effect on cooking loss. In addition, the epistatic locus on SSC16 (102 to 106 cM) did not only affect cooking loss. Influences on shear force were also detectable within an interaction between SSC2 (145 cM) and SSC16 (102 cM). The highest explained proportion of the phenotypic variance was 8.2% for IMF between SSC1 (263 cM) and SSC6 (101 cM) and 8.5% for an intrachromosomal epistatic QTL pair on SSC5.

## Discussion

Most QTL studies in pigs involve additive and dominance effects but epistasis is often ignored. To our knowledge, seven studies using epistatic models in pigs have been published [15-20,28]. In general, the use of epistatic models makes it possible to identify QTL, which interact with other QTL not only in an additive way but also via a × a, a × d, d × a and d × d interactions. In comparison to single- or double-QTL analyses, the main benefit of including epistatic QTL effects is the detection of novel QTL that affect a quantitative trait through epistatic interactions with another locus [4]. The identification of a considerable number of novel QTL in our study underlines this advantage. However, analyzing epistatic effects between two loci is computationally demanding because all pairwise combinations must be investigated [15,16]. In addition, the use of microsatellite information renders the distinction between two loci on the same or different chromosomes approximate.

In this study, 56 epistatic QTL pairs involving 104 interacting QTL positions were identified across all the autosomes for porcine carcass composition and meat quality traits. As shown in Tables 2 and Additional file 3 (see Additional file 3), 12 of these epistatic QTL positions were detected both in the single-QTL analysis of Liu et al. [23,31] and as novel epistatic QTL in our study. Six regions were related to carcass composition and six to meat quality traits. It can be assumed that these epistatic QTL play an important role in the expression of these phenotypes.

In regard to carcass composition (ECLC and fat muscle ratio), one epistatic QTL position located on SSC2 (125 to 135 cM) interacts with two other QTL regions on SSC4 (98 cM) and SSC7 (1 cM), respectively. This SSC2 locus was previously reported by Liu et al. [23] as a single QTL and by Lee et al. [32], who analyzed a Meishan × Pietrain cross. The same position was also detected for the loin eye area trait by Estelle et al. [33].

The epistatic relationships between SSC2 (125 to 135 cM) and regions on SSC4 (98 cM) and SSC7 (1 cM) explain 2.9% of the phenotypic variance for ECLC. The corresponding entire QTL variances (sum of epistatic and individual QTL variances) at these positions are 4.5% and 5% respectively, for the interactions between SSC2 (135 cM) and SSC4 (98 cM) and SSC2 (125 cM) and SSC7 (1 cM). It can be assumed that the 2% difference between epistatic and entire QTL variances is due to the individual QTL effect of the locus on SSC2, which was reported by Liu et al. [23]. It follows from this that the effects of the individual QTL loci on SSC4 and SSC7 are presumably small and difficult to detect in a single-QTL analysis. *Calpastatin *(*CAST*) and *tropomyosin *(*TPM4*) located on SSC2 between 125 and 135 cM are potential candidate genes for ECLC [34,35]. The locus on SSC4 (98 cM) is related to backfat and loin eye area traits [36-38]and carries the candidate gene *transforming growth factor beta-3 *(*TGF-β3*) [39]. In conclusion, all three genes play roles in skeletal, muscle and tissue development. The locus on SSC2 (125 cM) is also influenced by a region on SSC7 (1 cM) where Ponsuksili et al. [40] have identified a QTL for several backfat traits in a Duroc × Berlin Miniature pig F_2 _cross.

Additionally, we observed an interacting QTL pair between SSC8 (58 to 62 cM) and SSC10 (70 to 80 cM) that influences the loin eye area, ECLC and fat:muscle ratio traits. The involvement of the SSC8 locus had already been detected by a single-QTL analysis of these three traits [23]. For the fat:muscle ratio, the proportion of phenotypic variance was completely explained by epistatic effects. There was a 2% difference between epistatic variance and the sum of epistatic and individual QTL variances for the ECLC and loin eye area traits. Considering the single QTL variances presented by Liu et al. [23], we conclude that the SSC8 locus (58 to 62 cM) has important single QTL and epistatic QTL effects, whereas the SSC10 locus (70 to 80 cM) has only epistatic effects. This assumption is partially contradicted by Thomsen et al. [41], who has reported a single QTL at the same position on SSC10 that only affects the loin eye area trait.

In regard to the fat area trait, a region on the p arm of SSC6 (73 cM) interacts with SSC13 (11 cM), and a region on the q arm of SSC6 (113 cM) interacts with SSC12 (32 cM). The locus on the p arm of SSC6 has been previously detected by Liu et al. [31] and the locus on the q arm by Mohrmann et al. [42] in a resource family of Pietrain and crossbred dams (created from Large White, Landrace and Leicoma breeds). *Leptin receptor *(*LEPR*), which is involved in neonatal growth and development [43], is a candidate gene for the region on the SSC6 q arm.

A significant epistatic relationship was detected between SSC16 (80 cM) and SSC18 (21 cM) for BFT-13/14 rib. As shown by the QTL variance ratios in Table 2, this effect between both positions is mainly epistatic. However, Liu et al. [23] had identified the QTL region on SSC16 not for BFT-13/14 rib but for other backfat traits in the DuPi population. The locus on SSC18 was detected in the DuPi population by Edwards et al. [44] and in a cross of Berkshire and Yorkshire breeds [41]. Both studies included imprinting effects in the single-QTL models. Although Liu et al. [23] had applied a similar imprinting model, they did not identify an effect on SSC18 for backfat traits.

In this study, BFT thickness is influenced by an epistatic QTL pair on SSC7 (138 cM) and SSC13 (61 cM). The QTL position on SSC7 has not been identified as a single QTL in our population but it has already been reported in two studies [40,45]. Ponsuksili et al. [40] have shown that the region surrounding the locus on SSC7 is involved in the hepatic metabolic pathway.

Five epistatic QTL pairs involving ten loci were identified for pH 24 h in ham. Three QTL, located on SSC1 (108 cM), SSC2 (179 cM) and SSC15 (61 cM), have been previously detected by Liu et al. [23] in a single-QTL analysis and the QTL on SSC1 (108 cM) was shown to interact with a region on SSC5 (126 cM). Twelve percent of the phenotypic variance has been explained by this QTL pair, with 4% going back to the epistatic term and 8% to the single QTL on SSC1 reported by Liu et al. [23]. In addition to the work of Liu et al. [23], we analyzed the IMF and pH decline traits with a single-QTL model. No single QTL was found for IMF, whereas SSC15 (69 cM), which is comparable to the position detected for pH 24 h mentioned above, and SSC1 (119 cM) were identified for pH decline.

Furthermore, all these regions have been shown to carry several candidate genes involved in muscle development, composition and metabolism [46], e.g., *alpha-tropomyosin *(*TPM1*) and *ATP synthase, H+ transporting, mitochondrial F1 complex, alpha subunit 1 *(ATP5A1) related to the region on SSC1; and *myosin binding protein C *(*MYBPC1*) and *ATP synthase, H+ transporting, mitochondrial F1 complex *(*ATP5B*) related to SSC5 [47,48].

A position on SSC2 (145 to 166 cM) related to shear force is significant for individual and epistatic QTL effects [23] and has been identified in a Berkshire × Duroc intercross [49]. This region interacts with loci on SSC7, SSC13 and SSC16. The SSC7 and SSC13 loci have been described as single QTL in other studies [44,50,51]. A particularly large number of candidate genes has been identified for the epistatic relationship between SSC2 (166 cM) and SSC7 (87 cM). The SSC2 locus contains genes such as *tropomyosin-4 *(*TMP4*) and *GM2 activator protein *(*GM2A*) [52,53], whereas SSC7 carries the *myosin, heavy chain 6 *(*MYH6*) and *myosin, heavy chain 7 *(*MYH7*) genes [53]. The biological functions of these genes are primarily related to muscle composition.

Until now, we have only discussed epistatic QTL pairs with at least one locus previously detected as a single QTL in the DuPi population analyzed by Liu et al. [23]. We have identified many other epistatic loci that do not have a corresponding result in the single-QTL analysis. Of the 104 QTL positions involved in the 56 epistatic QTL, 12 have been reported by Liu et al. [23] and are detected by our single-QTL analysis, 30 have been reported in the literature and 62 are presumably novel positions. In general, the effects of these QTL pairs can be explained by purely epistatic effects, in which the single QTL of each involved position is of minor importance. The significance of the epistatic effects can be inferred from the difference between the epistastic variance and the sum of epistatic and individual QTL variances, which is frequently close to zero (Table 2). Similar results have been reported by Duthie et al. [16], who also detect novel QTL based on an epistatic QTL analysis.

Although many QTL have been reported in the literature (Table 3), we did not detect any single QTL for the IMF trait. Of particular relevance to this trait are the two epistatic QTL studies of Ovilio et al. [18] and Duthie et al. [16], which have revealed two epistatic QTL pairs related to loci on SSC1 and SSC4 and on SSC6 and SSC9. Here we identified four epistatic QTL loci on SSC1 (263 cM), SSC5 (87 cM) and SSC6 (101 cM). The QTL region detected on SSC1 was comparable to the identified epistatic QTL locus described by Duthie et al. [16] and to the individual QTL in other studies on this trait [44,54]. In other single-QTL studies, loci on SSC5 (87 cM) and SSC6 (101 cM) have been identified as influencing IMF [55,56].

**Table 3:** Reported QTL in the literature around similar locations as the QTL identified in the present study

Carcass composition	**SSC (position cM)**^**1**^	Flanking marker	**Reference**^**2**^
BFT 13/14 rib	18 (21)	SW2540 - SW1023	[41,44]

BFT shoulder	10 (151)	SW2067	[64]
	15 (84)	SW1119	[65]

BFT thickness	7(138)	S0101	[40,45]

ECLC	2 (135)	SW1564 - SW834	[23,66]
	8 (62)	SW1029 - SW7	[23]

Fat area	6 (112)	S0003	[31,42]

Fat muscle ratio	2 (125)	SW240 - SW1564	[23,32]
	8 (62)	SW1029 - SW7	[23]

Loin eye area	2 (135)	SW1564 - SW834	[33]
	4 (96)	S0214 - S0097	[37]
	8 (58)	SW1029 - SW7	[23,44,67]
	10 (70)	SW830 - S0070	[41]
	17 (55)	SW840 - SW2431	[68]

**Meat quality 1**	**SSC (position cM)**^**1**^	**Flanking marker**	**Reference**^**2**^

pH decline loin	3 (52)	SW2570 - S0002	[44]
	6 (39)	S0035 - S0087	[69]
	15 (61)	SW936 - SW1119	[69]

pH 24 h loin	3 (16)	SW27 - S0164	[18]
	4 (14)	S0227 - S0001	[50]
	10 (84)	S0070 - SW951	[59]
	11(39)	S0071 - S0009	[50]
	18 (24)	SW1023 - SB58	[51]

pH 24 ham	1 (108)	S0312 - SW2166	[23,54,70]
	2 (179)	SWR2157 - SW1879	[23,33]
	5 (126)	IGF1 - SW1954	[69,71]
	10 (84)	S0070 - SW951	[59]
	15 (61)	SW936 - SW1119	[31]
	18 (23)	SW1023 - SB58	[51]

Conductivity. 24 h loin	5 (52)	SWR453 - SW2425	[72]
	13 (75)	TNNC - SW398	[73,74]

Conductivity 24 h ham	10 (99)	S0070 - SW951	[31]

Meat color	7 (80)	SW175 - S0115	[18]

**Meat quality 2**	**SSC (position cM)**^**1**^	**Flanking marker**	**Reference**^**2**^

Cooking loss	7 (45)	S0025 - S0064	[50]
	13 (13)	S0219 - SW344	[58]
	15 (16)	S0355 - SW1111	[68]

Shear force	2 (150)	SW834 - S0226	[23,49]
	7 (87)	SW175 - S0115	[44,51]
	13 (112)	SW398 - S0289	[50]

IMF	1(263)	SW2512	[16,44,54]
	5 (87)	S0005 - SW1987	[56]
	6 (101)	S0059 - S0003	[55]

SSC *Sus scrofa *chromosome

^1 ^position of the QTL in cM

^2 ^references of other studies reporting QTL in similar regions of the specific chromosome

Significant epistatic relationships can be observed between QTL positions on SSC7, SSC13 and SSC16, which mainly influence the expression of cooking loss and shear force. A QTL locus on SSC7 (40 to 50 cM) for cooking loss has been reported by de Koning et al. [50] in an F_2 _cross of Meishan and commercial Dutch pigs and this region carries the *MHC *genes, which are potential candidate genes [57]. Other single-QTL analyses have revealed epistatic loci on SSC13 (13 cM) and SSC16 (108 cM) [31,58]. The epistatic QTL position on SSC16 (102 to 108 cM) also interacts with loci on SSC4 (43 cM, cooking loss) and SSC2 (145 to 160 cM, shear force). Though a novel QTL, SSC16 may play an important role in tenderness traits.

Three epistatic QTL pairs not yet mentioned are involved in the expression of loin pH 24 h. All the QTL positions involved have been reported in the literature and are relevant for meat quality [18,50,51,59]. Moreover, four QTL pairs involving eight epistatic QTL loci are relevant for loin pH 1 h. Although all the positions for this trait have not been published yet, many other loci are well known. The high number of epistatic interactions shows the complexity of postmortem metabolic processes in meat, which need further clarification [60]. As an example of this complexity, Figure 1 depicts all the epistatic loci for pH traits. Most QTL pairs have an impact on more than one trait, and the number of QTL positions that epistatically influence a single trait ranges from three to eight. Pleiotropy and co-regulation are important factors of genetic control to compensate for up- and down-regulation of correlated traits by gene interactions [8,61].

Epistasis appears to be an important contributor to genetic variation in carcass composition and meat quality traits. Subdividing epistatic effects into the structural types (a × a, a × d, d × a and d × d) allows a deeper insight into the genetic mechanisms behind the expression of these phenotypes. As shown in Additional file 2 (see Additional file 2), all types of structural epistasis can be found across all traits. Often, more than one component is significant, indicating complex genetic structures, particularly for meat quality traits. On average, d × d interactions are the most prevalent. Twenty-nine pairs exhibit d × d, 19 a × a, 11 a × d and 13 d × a epistatic effects. Moreover, the importance of dominance becomes more obvious by summing up the three epistatic effects (a × d, d × a and d × d) that comprise dominance. With respect to all traits, we observed this composite effect for 33 of 40 cases, which makes it more important than a × a effects. Epistatic dominance contributes to heterosis, and it has been widely shown that heterosis plays an important role in the genetics of carcass composition and meat quality [62].

For seven QTL pairs, a × a effects were more prevalent in the expression of traits (e.g., epistasis among SSC3 and SSC14 for conductivity 1 h loin) than were other interaction effects containing dominance. According to Carlborg and Haley [4], a × a effects are indicators of co-adaptive epistasis and occur when the homozygous alleles of the two loci that originate from the same parental line show enhanced performance. This type of gene interaction is particularly interesting, since the loci have no significant individual effects [4]. This might be the reason why some of our novel epistatic QTL positions have not been not found in a single-QTL analysis. Selection strategies among the parental lines might lead to fixation of different alleles at the relevant loci, regulating the expression of a specific phenotype in a way that makes statistical epistasis unapparent in either population [17].

## Conclusions

In the present study, a bi-dimensional scan identified a large number of epistatic QTL pairs involved in the expression of carcass composition and meat quality traits. These results show that the genetic architecture of carcass composition and meat quality is mainly composed of a complex network of interacting genes rather than of the sum of individual QTL effects. Combining epistatic QTL experiments with subsequent gene expression profiling can be a promising strategy to clarify the underlying biological processes of muscle development and metabolism.

## Competing interests

The authors declare that they have no competing interests.

## Authors' contributions

CG performed data analysis and drafted the manuscript. EJ and CP participated in the design of the study. HB helped in the statistical analysis and its assembly. HJ coordinated the collection of data. CL, DT and KS participated in the study's design and coordination and helped to draft the manuscript. ET conceived the study and participated in its design, coordination and statistical analysis and helped to draft the manuscript. All the authors have read and approved the final manuscript.

## Supplementary Material

Additional file 1 **Genetic markers used in this study**. For all genetic markers map positions, numbers of alleles and polymorphic information content (PIC) along with a corresponding PIC-plot are presented. Click here for file

Additional file 2 **Impact of epistatic effects for carcass composition and meat quality traits**. Individual and epistatic QTL effects subdivided into the underlying structural components are presented. Click here for file

Additional file 3 **Relevant single QTL identified in the study of Liu et al**. [23,31]**for carcass composition and meat quality traits**. The table contains the 12 corresponding QTL positions which were detected in the single QTL analysis of Liu et al. [23,31] and our epistatic QTL study. Click here for file
