# Supplementary material for: Epistatic QTL pairs associated with meat quality and carcass composition traits in a porcine Duroc × Pietrain population
Source: Genet Sel Evol. 2010 Oct 26;42(1):39. doi: 10.1186/1297-9686-42-39 (PMC2984386; doi:10.1186/1297-9686-42-39)
Supplement: Additional file 1 — Genetic markers used in this study. For all genetic markers map positions, numbers of alleles and polymorphic information content (PIC) along with a corresponding PIC-plot are presented. [file 1297-9686-42-39-S1.PDF]

Additional file 1 - Genetic markers used in this study

Table S1 - Genetic markers used in this study

SSC	Locus[1]	Position[2]	Allele	PIC[3]
1	SW1824	0.0	4	0.64
	SW1515	13.7	6	0.74
	SWR2300	28.5	4	0.57
	SW1851	38.0	4	0.67
	SW1653*	61.8	6	0.37
	SW952*	74.3	2	0.00
	S0312	93.5	5	0.78
	SWR702*	108.6	5	0.12
	SW2166	119.4	5	0.69
	S0113	119.6	2	0.04
	SWR2182*	151.1	4	0.17
	SWR982*	170.6	4	0.14
	S0155	178.5	4	0.98
	SW1311*	198.6	5	0.45
	AMBP*	214.5	2	0.00
	SW1957	237.0	5	0.54
	SW373	243.1	4	0.22
	SW1301	261.6	6	0.90
	SW2512	263.7	5	0.50
2	SW2443	0.0	4	0.43
	SW2623	12.9	5	0.52
	S0141	32.6	4	0.32
	FTH1*	67.2	2	0.00
	SW240	118.0	7	0.75
	SW1564	127.1	2	0.04
	SW834	142.6	8	0.89
	S0226	152.0	6	0.94
	SW1517	154.9	6	0.86
	SWR2157	168.7	6	0.77
	SW1879	180.4	4	0.47
	SW1844	191.3	3	0.77
	SWR308	206.8	7	0.78
3	SW72	0.0	5	0.85
	S0164	36.6	9	0.93
	SW2570	50.2	5	0.30
	S0002	86.7	5	0.54
4	S0227	0.0	2	0.41
	S0001	40.1	4	0.90
	S0214	66.3	6	0.72
	S0097	115.6	5	0.42
5	ACR	0.0	7	0.63
	SJ024*	0.5	6	0.36
	SW314	3.1	4	0.55
	SW491	16.3	1	0.00
	SW1482	29.5	4	0.90
	SWR453	47.2	3	0.58
	SW2425*	58.7	6	0.31
	S0092	61.7	6	0.88
	SW1134	71.5	2	0.05

SSC	Locus[1]	Position[2]	Allele	PIC[3]
	S0005	77.5	9	0.84
	SW1987	90.7	4	0.34
	IGF1	115.1	5	0.20
	SW1954	129.9	3	0.41
	SW378	138.2	2	0.01
	SW967	150.9	5	0.57
6	S0035	0.0	5	0.71
	S0087	61.2	4	0.81
	SW1067	70.6	5	0.98
	SW193	76.4	2	0.49
	S0300	77.5	3	0.64
	CKM*	80.6	2	0.00
	S0220	82.6	4	0.70
	S0059	99.3	5	0.50
	S0003	112.9	5	0.90
7	S0025	0.0	5	1.00
	S0064	33.0	6	0.75
	S0102	69.6	7	1.05
	SW175	79.6	4	0.95
	S0115	107.4	6	0.71
	S0101	138.2	4	0.83
8	SW2611	0.0	5	0.33
	S0353*	12.6	4	0.21
	SW905*	23.5	4	0.16
	KS195*	40.5	6	0.37
	SW1029*	54.1	2	0.01
	SW7*	68.4	5	0.38
	S0086	80.3	5	0.99
	SW2160*	98.4	3	0.28
	GC*	103.3	2	0.00
	S0144	108.2	3	0.32
	SW61	127.8	9	0.93
9	SW21	0.0	3	0.89
	SW911	19.4	5	0.57
	SW54	56.1	4	0.63
	S0109	68.0	2	0.25
	S0295	82.7	3	0.29
10	SW830	0.0	4	0.66
	S0070	83.7	7	1.00
	SW951	123.2	4	0.41
	SWR67	151.1	2	0.51
	SW2067	151.2	5	0.16
11	SW2008	0.0	4	0.72
	S0071	28.8	5	0.92
	S0009	38.6	4	0.62
	SW703	67.9	3	0.69
12	SW2490	0.0	6	0.98
	S0143	2.8	3	0.09
	SW874	65.4	5	0.67
	SW605	151.0	3	0.38
13	S0219	0.0	3	0.50
	SW344	56.4	5	0.48

SSC	Locus[1]	Position[2]	Allele	PIC[3]
	TNNC*	69.6	2	0.00
	SW398	100.9	5	0.96
	S0289	129.1	5	0.60
	S0215	129.2	2	0.01
14	SW857	0.0	5	0.70
	PPP*	30.0	2	0.00
	S0007	68.8	8	0.91
	SWC27	113.3	4	0.31
15	S0355	0.0	5	0.84
	SW1111	27.8	7	0.91
	SW936	60.6	5	0.76
	SW1119	84.1	5	0.69
16	S0111	0.0	6	0.84
	S0026	70.7	3	0.59
	S0061	108.0	4	0.65
17	SW335	0.0	3	0.71
	SW840	45.0	2	0.13
	SW2431	80.7	3	0.54
18	SY4*	0.0	4	0.46
	SW1808	8.5	5	0.58
	SW2540*	10.2	4	0.28
	SW1023	22.2	5	0.73
	SB58*	41.9	4	0.30
	SW787	43.2	5	0.94
	S0062	56.9	3	0.25
	SW1682*	58.4	4	0.35
	S0120*	61.5	4	0.35
	SJ061*	64.1	4	0.17
	SWR414*	81.2	4	0.97
	SY31*	94.4	3	0.07

SSC *Sus scrofa* chromosome

[1] additional included genetic markers are marked with * in comparison to the study of Liu et al. (2007)

[2] position on genetic map in Kosambi cM

[3] polymorphic information content

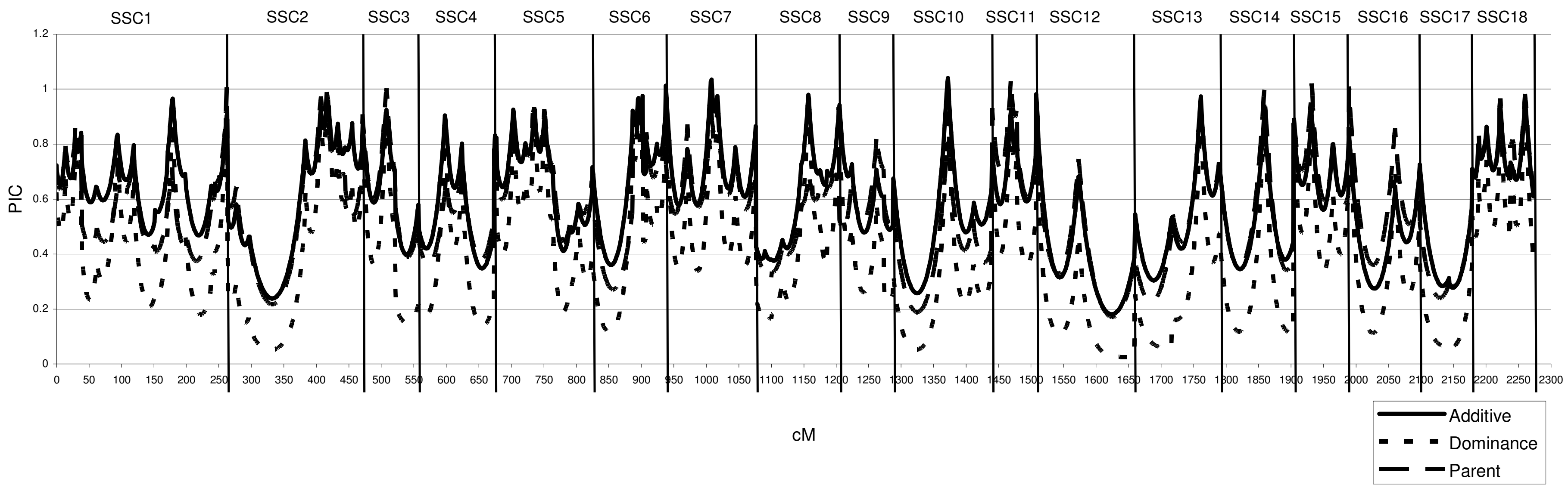

Figure S1 – PIC-plot of genetic markers used in this study

SSC: *Sus scrofa* chromosome
