# Supplementary material for: Epistatic QTL pairs associated with meat quality and carcass composition traits in a porcine Duroc × Pietrain population
Source: Genet Sel Evol. 2010 Oct 26;42(1):39. doi: 10.1186/1297-9686-42-39 (PMC2984386; doi:10.1186/1297-9686-42-39)
Supplement: Additional file 2 — Impact of epistatic effects for carcass composition and meat quality traits. Individual and epistatic QTL effects subdivided into the underlying structural components are presented. [file 1297-9686-42-39-S2.PDF]

Additonal file 2 – Impact of epistatic effects for carcass composition and meat quality traits

**Carcass composition**	**SSC pos. 1 (cM)**[1]	**SSC pos. 2 (cM)**[1]	**a_1**[2]	**d_1**[2]	**a_2**[2]	**d_2**[2]	**$I_{a\times a}$**[3]	**$I_{a\times d}$**[3]	**$I_{d\times a}$**[3]	**$I_{d\times d}$**[3]	**SE range**[4]
BFT 13/14 rib	16 (80)	18 (21)	-0.01	-0.02	0.09	-0.08	-0.11	-0.07	**-0.18**	0.14	0.03 – 0.04
BFT shoulder	2 (207)	15 (84)	0.08	0.09	0.15	0.04	**0.21**	0.00	**-0.32**	**-0.23**	0.06 – 0.20
	9 (57)	10 (151)	0.02	0.19	0.08	**0.23**	**0.24**	-0.05	-0.10	**-0.38**	0.06 – 0.33
BFT thickness	7 (138)	13 (61)	-0.12	0.11	0.07	-0.04	**-0.14**	**0.27**	-0.02	**-0.21**	0.04 – 0.14
Dressing	5 (1)	9 (15)	-0.23	-0.08	-0.16	-0.51	-0.72	0.12	-0.11	0.32	0.17 – 0.41
ECLC	2 (135)	4 (98)	-0.12	**-7.34**	-1.87	**-7.57**	2.07	-0.99	3.53	**15.47**	0.66 – 6.12
	2 (125)	7 (1)	-0.70	1.35	0.68	0.49	0.83	0.40	-1.76	-1.25	0.28 – 0.86
	8 (62)	10 (79)	-0.33	1.62	-0.60	2.58	0.32	1.75	1.37	-5.20	0.36 – 1.39
Fat area	6 (113)	12 (32)	0.29	0.82	-0.93	0.78	-0.71	-1.22	2.10	-2.00	0.36 – 1.37
	6 (73)	13 (11)	-0.39	0.88	-0.29	1.26	0.33	0.39	1.56	-2.09	0.25 – 0.70
	8 (36)	8 (127)	-0.72	-2.09	0.20	-2.31	0.60	1.65	-1.13	**4.14**	0.42 – 1.14
Fat muscle ratio	2 (125)	7 (1)	0.02	-0.04	-0.02	-0.02	-0.03	-0.01	**0.06**	**0.05**	0.01 – 0.02
	8 (62)	10 (80)	0.01	-0.04	0.02	-0.05	-0.01	-0.04	**-0.05**	**0.12**	0.01 – 0.04
	8 (80)	17 (45)	-0.04	**-0.06**	0.01	-0.03	0.01	**0.06**	-0.03	**0.15**	0.01 – 0.05
Loin eye area	2 (135)	4 (96)	0.27	**-17.51**	-1.47	**-16.54**	4.30	-2.72	3.86	**36.80**	1.28 – 12.07
	8 (58)	10 (70)	-2.21	5.50	-0.54	**6.81**	0.10	7.19	1.97	**-14.98**	0.88 – 2.34
	17 (55)	17 (80)	-3.34	-11.79	6.55	-9.84	**-14.58**	8.62	**-15.32**	4.10	2.78 – 9.85
**Meat quality 1**	**SSC pos. 1 (cM)**[1]	**SSC pos. 2 (cM)**[1]	**a_1**[2]	**d_1**[2]	**a_2**[2]	**d_2**[2]	**$I_{a\times a}$**[3]	**$I_{a\times d}$**[3]	**$I_{d\times a}$**[3]	**$I_{d\times d}$**[3]	**SE range**[4]
pH 1h loin	2 (156)	18 (9)	0.00	**0.06**	0.05	0.03	**-0.11**	0.02	**-0.09**	-0.05	0.02 – 0.07
	3 (34)	13 (85)	**0.06**	**0.09**	**-0.08**	**0.11**	0.03	**-0.09**	**0.20**	**-0.17**	0.03 – .011
	8 (1)	15 (77)	0.00	0.04	0.03	0.04	**0.07**	-0.02	0.01	**-0.11**	0.02 – 0.05
	12 (45)	16 (1)	-0.04	**-0.09**	-0.02	**-0.08**	**-0.12**	0.02	0.02	**0.17**	0.02 – 0.05
pH 24h loin	3 (16)	11 (39)	-0.01	-0.02	-0.01	-0.02	**-0.03**	0.02	**0.03**	**0.06**	0.01 – 0.02
	4 (14)	11 (16)	0.02	**0.05**	**-0.06**	**0.04**	0.01	-0.05	**0.12**	-0.08	0.01 – 0.04
	10 (84)	18 (24)	0.02	0.01	-0.01	-0.02	**-0.04**	**-0.04**	0.00	-0.02	0.01 – 0.02
pH decline loin	3 (13)	6 (41)	-0.01	-0.03	**0.08**	0.00	0.00	**0.07**	**-0.24**	0.06	0.03 – 0.11
	3 (52)	18 (22)	0.03	**-0.12**	0.01	**-0.06**	**-0.09**	-0.01	-0.01	**0.20**	0.02 – 0.07
	6 (39)	14 (84)	**0.11**	**-0.26**	-0.01	**-0.28**	**0.08**	**-0.32**	0.00	**0.57**	0.05 – 0.22
	8 (6)	15 (71)	0.02	**0.09**	0.04	**0.08**	**0.09**	-0.03	0.01	**-0.18**	0.03 – 0.07
	12 (48)	16 (1)	-0.05	-0.10	-0.02	-0.09	-0.13	0.04	0.01	0.18	0.02 – 0.08
	15 (61)	17 (29)	0.10	0.03	0.00	0.01	**-0.14**	**-0.11**	-0.02	-0.04	0.03 – 0.12

pH 24h ham	1 (108)	5 (126)	-0.01	-0.02	**0.05**	**0.03**	0.02	**-0.10**	**-0.09**	0.00	0.02 – 0.05
	2 (179)	7 (122)	0.04	**-0.14**	0.01	**-0.10**	**0.06**	-0.02	-0.02	**0.20**	0.02 – 0.08
	7 (88)	12 (1)	0.01	-0.02	0.01	-0.02	**0.05**	-0.01	0.00	**0.06**	0.01 – 0.03
	10 (84)	18 (23)	0.01	-0.01	0.00	-0.05	**-0.07**	-0.02	0.00	0.03	0.01 – 0.05
	15 (61)	18 (92)	0.02	**0.08**	-0.01	**0.07**	**-0.03**	**-0.07**	**-0.03**	**-0.15**	0.01 – 0.04
Conductivity 1h loin	3 (10)	14 (113)	0.01	-0.34	0.09	0.12	**-0.59**	-0.15	-0.24	**0.39**	0.12 – 0.69
Conductivity 24h loin	5 (52)	13 (75)	-0.06	**0.99**	0.11	1.61	-0.36	0.11	-0.42	**-2.17**	0.11 – 0.35
	6 (13)	13 (20)	-0.05	0.01	-0.34	0.20	-0.34	-0.15	0.59	0.17	0.11 – 0.39
Conductivity 24h ham	10 (99)	13 (30)	1.05	1.77	-0.39	1.87	-1.37	-2.93	0.41	**-3.31**	0.34 – 2.62
Meat color	7 (80)	12 (26)	1.16	3.95	-1.15	3.93	3.27	-1.98	0.67	**-7.93**	0.77 – 0.03

**Meat quality 2**	**SSC pos. 1 (cM)**[1]	**SSC pos. 2 (cM)**[1]	a_1[2]	d_1[2]	a_2[2]	d_2[2]	$I_{a\times a}$[3]	$I_{a\times d}$[3]	$I_{d\times a}$[3]	$I_{d\times d}$[3]	**SE range**[4]
Cooking loss	1 (97)	16 (63)	-0.69	-0.33	-0.33	0.03	0.95	1.58	1.14	0.36	0.31 – 0.85
	2 (186)	15 (16)	-0.94	-0.38	-0.26	-0.57	-0.85	**2.20**	0.78	1.11	0.36 – 2.20
	4 (43)	16 (102)	-0.37	0.34	0.57	-1.20	1.42	0.71	-0.52	1.48	0.33 – 1.21
	5 (4)	18 (82)	0.73	0.07	0.02	0.28	-0.82	-0.75	0.10	0.03	0.21 – 1.77
	7 (50)	13 (13)	-0.10	1.07	-0.54	0.84	0.68	1.24	0.74	**-2.24**	0.29 – 0.97
	7 (47)	16 (108)	-0.20	0.08	0.67	-0.87	-0.33	1.81	-0.85	0.75	0.26 – 0.76
	7 (40)	17 (60)	-1.55	-1.96	-2.01	**-2.78**	0.76	**3.99**	**4.04**	**3.53**	0.53 – 2.98
	8 (85)	18 (8)	-0.57	0.45	-0.24	0.80	0.66	1.42	-0.32	-2.02	0.23 – 0.62
Thawing loss	2 (49)	4 (105)	-1.18	**-4.01**	0.48	-3.23	**-4.04**	2.73	0.28	**5.07**	0.91 – 11.35
	15 (8)	17 (1)	-0.15	0.07	-0.55	-0.06	-0.37	0.51	1.15	0.93	0.21 – 0.51
Shear force	2 (166)	7 (87)	-0.07	-6.83	-1.44	-4.38	-3.05	4.00	4.82	9.59	1.07 – 4.39
	2 (150)	13 (112)	2.68	-11.46	-0.14	-10.81	2.54	-1.28	2.49	**23.60**	1.23 – 6.34
	2 (145)	16 (102)	1.96	8.43	-2.43	7.31	-0.93	-0.36	6.35	**-16.98**	1.09 – 4.11
	8 (84)	8 (111)	-1.41	1.22	1.55	1.93	-3.37	0.06	-0.80	**-7.77**	1.90 – 3.74
IMF	1 (263)	6 (101)	-0.35	-0.54	-0.84	-0.65	1.27	-0.12	1.77	0.53	0.33 – 1.00
	5 (57)	5 (87)	-2.74	0.74	2.69	-0.85	0.53	4.01	-4.28	0.82	0.65 – 1.15

SSC *Sus scrofa* chromosome

[1] position of QTL in Kosambi cM

[2] estimated additive (a) and dominance (d) effects for individual QTL related to position 1 or position 2

[3] estimated additive × additive ($I_{a\times a}$), additive × dominance ($I_{a\times d}$), dominance × additive ($I_{d\times a}$) and dominance × dominance ($I_{d\times d}$) effects; prevalent epistatic or individual effects which are twice the residual variance of the phenotypic trait are presented in bold

[4] SE standard error ranges for all genetic effects of one epistatic QTL
