# Supplementary material for: Epistatic QTL pairs associated with meat quality and carcass composition traits in a porcine Duroc × Pietrain population
Source: Genet Sel Evol. 2010 Oct 26;42(1):39. doi: 10.1186/1297-9686-42-39 (PMC2984386; doi:10.1186/1297-9686-42-39)
Supplement: Additional file 3 — Relevant single QTL identified in the study of Liu et al. [23,31]for carcass composition and meat quality traits. The table contains the 12 corresponding QTL positions which were detected in the single QTL analysis of Liu et al. [23,31] and our epistatic QTL study. [file 1297-9686-42-39-S3.PDF]

Additional file 3 – Relevant single QTL identified in the study of Liu et al. [23, 31] for carcass composition and meat quality traits

SSC[1]	Trait[2]	F-ratio[3]	Pos.[4]	Flanking markers	Add.[5]	Dom.[5]	SE[6]	Vari.[7]
1	pH 24 h ham	24.66***	55.2	S0312-S0113	0.05	-0.02	0.01	9.08
2	pH 24 h ham	7.46*	61.8	SW1564-S0226	-0.02	-0.02	0.01	2.94
2	shear force	6.53*	65.5	SW834-S0226	-1.82	-0.51	0.62	4.52
2	fat muscle ratio	8.48**	54.6	SW2443-SWR308	-0.01	0	0.00	2.9
2	ECLC	9.67**	55.2	SW2623-SWR308	0.63	0.22	0.20	3.31
6	fat area[8]		35	S0035-S0087				6.60
8	loin eye area	9.49**	86.5	SW2611-S0144	-1.24	-0.87	0.42	3.23
8	fat muscle ratio	6.24*	86	S0086-S0144	0.01	0.01	0.01	2.15
8	ECLC	7.22*	86	S0086-S0144	-0.53	-0.42	0.21	2.5
10	cond. 24 h ham[8]		156	S0070-SW951				2.25
15	pH 24 h ham	5.86*	52.5	SW1111-SW1119	0.03	0.01	0.01	2.32
15	***pH dec loin***	***5.09****	***69***	***SW936-SW1119***	***-0.05***	***-0.01***	**0.02**	***1.37***

[1] SSC *Sus scrofa* chromosme
[2] line in bold: extended results for pH decline
[3] three significant levels were used: 5% chromosome wide significant level, i.e. suggestive level (*); 5% genome-wide significant level (F = 8.02 **); and 1% genome-wide significant level (F=9.76 ***)
[4] position in Kosambi cM
[5] add: additive effects, dom: dominance effects
[6] the average of the standard error (SE) for additive and dominance effects
[7] proportion of phenotypic variance explained by a QTL as a percentage of the residual variance in the F_2 population
[8] these QTL were identified by Liu et al. 2008 [31] using a combined line cross and half-sib analysis; therefore F-statistic and genetic values are missing
